# Do different lipid emulsions affect brain development? A single-center retrospective analysis

**DOI:** 10.3389/fnut.2026.1723062

**Published:** 2026-02-04

**Authors:** Samuele Caruggi, Andrea Calandrino, Benedetta Corradi, Marcella Battaglini, Paolo Massirio, Francesco Vinci, Federica Mongelli, Alessia Pepe, Alessandro Parodi, Mariya Malova, Agata Zoia, Mariasavina Severino, Martina Resaz, Andrea Rossi, Luca Antonio Ramenghi

**Affiliations:** 1 Neonatal Intensive Care Unit, Department of Maternal and Neonatal Health, IRCCS Istituto Giannina Gaslini, Genoa, Italy; 2 Department of Neuroscience, Rehabilitation, Ophthalmology, Genetics, and Mother/Child Sciences (DINOGMI), University of Genoa, Genoa, Italy; 3 Neuroradiology Unit, Department of Services, IRCCS Istituto Giannina Gaslini, Genoa, Italy; 4 Department of Health Sciences (DISSAL), University of Genoa, Genoa, Italy

**Keywords:** lipid emulsions, MRI, neurodevelopment, parenteral nutrition, preterm

## Abstract

**Background:**

Preterm birth is a critical period for brain development, as extrauterine factors can impair growth and myelination, thereby increasing the risk of neurodevelopmental impairment. Adequate nutrition and rapid weight gain are associated with better cognitive outcomes, and the choice of lipid emulsion during parenteral nutrition may influence these results. SMOFlipid®, enriched with omega-3 long-chain polyunsaturated fatty acids, could reduce inflammation and oxidative stress, potentially lowering bronchopulmonary dysplasia (BPD) risk. This study compared brain maturation at term-equivalent age (TEA) using MRI and neurodevelopment at 2 years in infants receiving either SMOFlipid® or Intralipid®.

**Methods:**

In this single-center retrospective observational cohort study, we included all very low birth weight (VLBW) infants admitted to the NICU of our institution between 2017 and 2021. Infants who underwent brain MRI at term-equivalent age and completed neurodevelopmental assessment at 2 years were included, and those with severe brain lesions were excluded. Patients were categorized into two groups based on the lipid emulsion administered during parenteral nutrition. The primary outcome was neurodevelopment at 24 months of corrected age (CA). The secondary outcome was brain maturation assessed by the total maturation score (TMS) on magnetic resonance imaging (MRI).

**Results:**

A total of 121 VLBW infants were included and categorized into two cohorts based on the lipid formulation administered: multi-component lipid emulsion (MLE) in 62 and soybean lipid emulsion (SLE) in 49 infants. TMS showed non-statistically significant differences among infants treated with SLE compared with those treated with MLE, as well as in neurodevelopmental outcomes assessed using Griffith’s scales.

**Conclusion:**

Despite integrating brain imaging and clinical follow-up data, this study could not determine the optimal lipid emulsion for preterm infants.

## 1 Introduction

Preterm infants are born during a critical period of brain development, making them particularly vulnerable to brain injury. Diffuse white matter injury (WMI) is currently recognized as the most common lesion associated with subsequent neurodevelopmental impairment (1–3).

The hallmark neuropathological feature of diffuse WMI is oligodendroglial lineage dysmaturation, mediated by oxidative stress that blocks oligodendrocyte progenitor cell maturation and myelination (1, 4, 5).

Although emerging research suggests a potential protective role of specific antioxidants—administered either *in vitro* or through the maternal diet—no clinical treatment is currently available to counteract these conditions, and it remains unclear whether the extrauterine environment can provide stimuli comparable to those *in utero* to support normal brain maturation (6–9).

Parenteral nutrition is a cornerstone in the management of very low birth weight (VLBW) infants, due to their intestinal immaturity and limited tolerance to enteral feeding (10). Identifying the optimal composition of parenteral nutrition, especially regarding lipid emulsions, is crucial to improving long-term health and quality of life in this vulnerable population. Several studies have highlighted the importance of optimal nutrition management to ensure faster weight gain from birth to term-equivalent age, which is subsequently associated with better neurodevelopmental outcomes in children born preterm (10–14).

In VLBW infants requiring parenteral nutrition with specific nutritional requirements, very long-chain omega-3 polyunsaturated fatty acids (PUFAs) are regarded as conditionally essential, with critical roles in early brain development and anti-inflammatory effects. Because of these factors, the choice of lipid emulsion in parenteral nutrition may influence clinical outcomes in neonates (15–17).

The influence of lipid formulations in parenteral nutrition on preterm neurodevelopment remains debated (18). SMOFlipid is a lipid emulsion combining soybean oil, medium-chain triglycerides (MCT), olive oil, and fish oil (60%:30%:25%:15%), providing a balanced fatty acid profile that includes eicosapentaenoic acid (EPA) and docosahexaenoic acid (DHA), with added α-tocopherol for antioxidant protection. Although multicomponent lipid emulsions such as SMOFlipid® have been associated with improved brain growth (16), better white matter organization (19), and anti-inflammatory effects (20–22) compared to pure soy bean oil emulsions, evidence from randomized controlled trials (RCTs) remains limited. To date, two RCTs (17, 23) have failed to demonstrate a clear neurodevelopmental advantage of SMOFlipid® over traditional soybean-based emulsions (Intralipid®), despite early signals of benefit such as enhanced head circumference growth and accelerated brain maturation (24, 25). However, other recent evidence from large cohort studies suggests potential benefits of SMOFlipid® over other lipids emulsions. Torgalkar (26) reported that SMOFlipid® was associated with significantly lower odds of any neurodevelopmental impairment in extremely preterm infants (<29 weeks’ gestation), and Chen (27) demonstrated that SMOFlipid® use was associated with a reduced prevalence of epilepsy, cerebral palsy, language delay and autism spectrum disorder at 2 and 5 years of age, particularly in VLBW infants. These findings suggest that, while biologically plausible, the superiority of fish oil-containing emulsions for long-term neurodevelopment in preterm infants remains unproven, underscoring the need for larger, well-powered studies to confirm their potential role in optimizing neurodevelopmental outcomes.

This study aims to provide new insights into the effects of parenteral lipid emulsions on neurodevelopment in preterm infants by comparing brain morphological maturation at term-equivalent age—assessed using magnetic resonance imaging (MRI)—and neurodevelopmental outcomes at 2 years of corrected age between two different lipid emulsions: a multicomponent lipid emulsion (MLE) (SMOFlipid® 20%, Fresenius Kabi, Italy) and a soybean-based lipid emulsion (SLE) (Intralipid® 20%, Fresenius Kabi, Italy).

## 2 Materials and methods

Preterm infants admitted to the Neonatal Intensive Care Unit of the IRCCS Giannina Gaslini Institute in Genoa, who underwent MRI at term-equivalent age (TEA) between 2017 and 2021, received two different lipid solutions, and completed a follow-up at 2 years of corrected age (CA), were retrospectively searched and included.

The primary outcome was the evaluation of neurodevelopment outcomes at 24 months of CA.

The secondary outcome was brain maturation assessed by MRI at TEA. We retrospectively included only VLBW infants who underwent MRI at TEA and who were evaluated at 24 months of CA by an expert pediatric neurologist using the Griffiths Scales of Child Development (GMDS) (0–2 years) (28).

The exclusion criteria were major cerebral malformations, brain lesions related to prenatal infection (e.g., from cytomegalovirus); metabolic diseases; arteriovenous malformation; perinatal stroke; and severe brain injury, including grade II and III intraventricular hemorrhage (IVH) according to Papile (29), post-hemorrhagic hydrocephalus (PHVD), periventricular hemorrhagic infarction (PVHI), any grade of periventricular leukomalacia (PVL) including periventricular punctate white matter lesions and any form of cerebellar hemorrhage (CBH)—all lesions with undoubted adverse effects on follow-up. Patients lost to follow-up before 24 months of correct age evaluation were excluded, as were patients whose MRI scans were deemed insufficient for TMS analysis.

In our NICU, the type of lipid emulsion used was changed from Intralipid to SMOFlipid following an institutional decision as part of company policy. Since January 2019, our NICU switched from Intralipid® to SMOFlipid®. Consequently, all parenteral nutrition administered from that date onward contained SMOFlipid® instead of Intralipid®, as these lipid emulsions are an integral component of parenteral nutrition.

According to this, the selected patients were then categorized into two cohorts:

1. Patients born between 2017 and December 2018 (inclusive) who received Intralipid®, an SLE.
2. Patients born from January 2019 to December 2021 who received SMOFLipid®, an MLE.

For each patient, information on pregnancy, delivery, and clinical course was collected. The need and duration of invasive mechanical ventilation, surfactant administration, bronchopulmonary dysplasia (BPD), according to NICHD 2001 (30), sepsis confirmed by blood culture or liquor culture, necrotizing enterocolitis (NEC), according to Bell (31), retinopathy of prematurity (ROP), duration of parenteral nutrition, and length of stay were assessed for every patient included.

Each VLBW infant underwent a brain MRI at TEA (between 39 and 41 weeks of CA) according to our internal protocol; MRI scans included axial T2- and T1-weighted images; 3 mm thick, T2-weighted coronal images; sagittal T1-weighted images; axial diffusion-weighted (*b*-value: 1,000 s/mm^2^); axial susceptibility weighted imaging (SWI); and diffusion tensor imaging (DTI).

The total maturation score (TMS) (32) was calculated for each patient who underwent MRI at term-equivalent age. TMS consists of four parameters obtained by MRI examination: myelination (M, 1–7 points), cortical convolutions (C, 1–6 points), germinal matrix (GM, 1–4 points), and bands of migrating glial cells (B, 1–4 points). TMS ranges from 4 to 21 points.

Brain MRI images were independently reviewed by two neonatologists, each with over 5 years of clinical experience, including specific expertise in neonatal neuroimaging and neuroradiological interpretation. The TMS was assigned according to standardized criteria, and discrepancies were resolved by consensus. The presence of posterior limb of internal capsule (PLIC) was also assessed in T1 and T2 MRI sequences and classified as usual (2 points), equivocal (1 point), and absent (0 points) (33).

After discharge from our NICU, all VLBW infants were enrolled in the anthropometric and neurodevelopmental follow-up program, which continued until they reached pre-school age. We retrospectively collected data from Griffiths Scales of Child Development (GMDS) (0–2 years) assessment performed at 24 months of correct age. GMDS yields five subscales: locomotor, personal–social, hearing and speech, eye and hand coordination, and performance. Each domain generates a standardized score, and a general developmental quotient (GDQ) is calculated by combining all subscale scores. The mean GDQ is 100 (SD ± 12), with higher scores indicating better developmental performance. Cognitive outcomes were classified as normal when GDQ was >85, borderline when GDQ ranged from 70 to 85, and delayed when GDQ was lower than 70 (28).

Statistical analyses were performed using Jamovi, an open-source statistical software based on the R language. Based on the variability observed in Gallini (17), the sample size required to detect clinically relevant differences in GDQ at 24 months of corrected age was estimated. Assuming a two-sided *α* of 0.05, 80% power, and equal allocation between groups, the required sample size varies according to the expected effect size: for a 5-point difference, approximately 253 infants per group (total ≈ 506) are required; for a 10-point difference, the required sample size decreases to 64 infants per group (total ≈ 128). Continuous variables were tested for normality and summarized as medians with interquartile ranges. We checked normality using the Shapiro–Wilk test and plots, but since most variables were not normally distributed, we used non-parametric methods such as the Mann–Whitney *U* test for analysis.

Inter-rater reliability for MRI evaluations was assessed using Cohen’s kappa coefficient. Group comparisons for continuous variables were conducted using the Mann–Whitney *U* test, while categorical variables were analyzed using the chi-square test or Fisher’s exact test, as appropriate. A *p*-value of <0.05 was considered statistically significant. To account for the increased risk of false positives due to multiple comparisons, the Benjamini–Hochberg procedure was applied to control the false discovery rate (FDR). This approach allows for a more balanced interpretation of statistical significance, particularly in exploratory analyses involving numerous *p*-values.

## 3 Results

During the study period, 933 neonates admitted to our NICU underwent brain MRI. Of these, 298 were VLBW infants. We excluded neonates with prenatal hemorrhagic lesions, congenital malformations, or congenital infections (*n* = 13), along with 47 VLBW infants with white matter lesions (23 in the Intralipid® group and 25 in the SMOFLipid® group), 16 with cerebellar hemorrhage of any grade, and 23 with grade II and III IVH, PHVD, or PVHI, resulting in a total of 123 excluded patients. Among the remaining 199 infants, in 41 patients, the MRI performed was not considered to be of sufficient quality for accurate TMS analysis and was therefore excluded, and in 37 cases, data were incomplete or the Griffiths assessment scheduled at 24 months of corrected age was not performed.

The two cohorts were composed as follows: 59 patients born between 2017 and December 2018 (inclusive) were included in the SLE group and 62 patients born from January 2019 (inclusive) to December 2021 (inclusive) were included in MLE group.

The two-cohort comparison showed no statistically significant differences in anthropometric characteristics (birth weight and gestational age), maternal characteristics (artificial insemination, twin birth, intrauterine growth retardation, preeclampsia, placental abruption, twin-to-twin transfusion syndrome, and complete corticosteroid prophylaxis). Clinical outcomes were analyzed across the two groups (Table 1).

**Table 1:** Clinical characteristics of the two cohorts.

Patients’ characteristics	INTRALIPID®	SMOFLIPID®	*p*-value	Adjusted *p*-value
Number of patients	59	62		
Gestational age at birth (median, IQR, and weeks)	29.5 (3.0)	29.8 (2.8)	0.43	0.65
Birth weight (median, IQR, and grams)	1,160 g (335)	1,190 g (318)	0.52	0.75
Medically assisted reproduction	18.4%	14.5%	0.59	0.76
Twin birth	42.9%	35.5%	0.43	0.65
Intrauterine growth retardation	26.5%	27.4%	0.91	0.91
Preeclampsia	16.3%	25.8%	0.22	0.50
Placental abruption	8.2%	9.7%	0.78	0.87
Twin-to-twin transfusion syndrome	8.1%	11.3%	0.58	0.76
Bronchopulmonary dysplasia (percentage)	41.1%	22.50%	**0.04**	0.26
Culture-proven sepsis (percentage)	34.60%	25.50%	0.17	0.50
Treated retinopathy of prematurity (percentage)	5.10%	9.20%	0.17	0.50
Necrotizing enterocolitis (percentage)	4.10%	3.20%	0.81	0.87
Germinal matrix hemorrhage (percentage)	6.7%	7.9%	0.84	0.87
Parenteral nutrition length (median, IQR, days)	17 (10)	21 (14)	0.18	0.50
Length of stay (median, IQR, and days)	46 (16)	49 (19)	0.23	0.50

Significant *p*-values are reported in bold.

The incidence of BPD was higher in the Intralipid® group compared to the SMOFLipid® group (41% vs. 22.5%) but was not statistical significant after Benjamini–Hochberg correction with an adjusted *p*-value of 0.26. No statistically significant differences were observed in the rates of culture-proven sepsis (34.6% vs. 25.5%, adjusted *p* = 0.5), treated retinopathy of prematurity (5.1% vs. 9.2%, adjusted *p* = 0.5), or necrotizing enterocolitis (4.1% vs. 3.2%, adjusted *p* = 0.87). Similarly, the duration of parenteral nutrition (median [IQR] 17 (11) vs. 21 (14) days, adjusted *p* = 0.5) and length of hospital stay (median [IQR] 46 (16) vs. 49 (19) days, adjusted *p* = 0.5) did not differ significantly between the two groups. The incidence of germinal matrix hemorrhage was comparable between the two groups, with 6.7% in the Intralipid® group and 7.9% in the SMOFLipid® group (*p* = 0.87), indicating no statistically significant difference.

The two cohorts were then compared regarding TMS using the Griffiths scales (Table 2).

**Table 2:** MRI and Griffith’s outcomes.

Patients’ characteristics	INTRALIPID®	SMOFLIPID®	*p*-value	Adjusted *p*-value
Number of patients	59	62		
Age at MRI (median, IQR, and weeks)	40.3 (2.3)	39.9 (1.7)	0.18	0.50
Weight at MRI (median, IQR, and grams)	2,970 g (725)	2,870 g (700)	0.24	0.50
Z-Score weight at MRI (median)	−1,13	−1,10	0.24	0.50
Total maturation score (median and IQR)	13 (3)	12 (2)	**0.007**	0.09
PLIC T1 (median)	2	2	0.184	0.50
PLIC T2 (median)	2	1	**0.005**	0.07
Griffiths at 2 years correct age (median and IQR)				
Total score	92 (9)	92 (8)	0.71	0.87
Locomotor (A)	90 (12)	92.5 (8)	0.27	0.50
Personal social (B)	95 (9)	96 (2)	0.27	0.50
Hearing and speech (C)	91 (11)	90 (13)	0.34	0.58
Eye and hand coordination (D)	85 (16.5)	93 (12)	**<0.001**	**0.02**
Performance (E)	92 (15)	88 (12)	0.74	0.87

PLIC, posterior limb internal capsula; MRI, magnetic resonance imaging. Significant *p*-values are reported in bold.

The Intralipid® group showed a higher TMS on MRI at term-equivalent age (median 13 vs. 12, adjusted *p* = 0.09), with a difference in PLIC visibility on T2-weighted sequences (median 2 vs. 1, adjusted *p* = 0.07). In contrast, no difference was found on T1-weighted sequences (adjusted *P* 0.5). The inter-rater agreement for MRI assessments was high, with a Cohen’s kappa of 0.87, reflecting almost perfect concordance between the two evaluators. At 2 years of corrected age, Griffiths III developmental scores were similar between groups across most domains: total score (92 vs. 92, *p* = 0.87), locomotor (90 vs. 92.5, *p* = 0.5), personal social (95 vs. 96, *p* = 0.5), hearing and speech (91 vs. 90, *p* = 0.58), and foundations of learning (92 vs. 88, *p* = 0.87). A statistically significant difference was observed only in the eye and hand coordination domain, where the SMOFLipid**®** group had higher scores (median 93 vs. 85, adjusted *p* = 0.02).

## 4 Discussion

Unlike the majority of previous studies that focused solely on clinically assessed neonatal outcomes (17, 23, 34), this research incorporated a validated morphological neuroimaging assessment using TMS, aiming to provide a more comprehensive and objective evaluation of brain morphological development (35–40). When focusing on lipids, the evaluation becomes particularly intriguing to examine the stages of myelination, as visualized on MRI.

At first glance, infants treated with Intralipid® showed higher TMS scores compared to those receiving SMOFLipid® (median 13 vs. 12); similarly, a more detailed analysis of the TMS distribution revealed slightly higher signal intensity in the PLIC on T2-weighted sequences in the Intralipid® group, but no differences were found on T1-weighted sequences, where myelination appear earlier than on T2-weighted images (41–43). PLIC plays a crucial role in routine clinical practice, as neuroradiologists often rely on its appearance to assess the normality of brain MRIs in term-equivalent neonates. Moreover, PLIC myelination may serve as a potential marker of nutritional quality during pregnancy in the offspring (44). However, after applying Benjamini–Hochberg correction for multiple comparisons, the statistical significance was lost, suggesting that this finding may be due to chance.

Neurodevelopmental outcomes, assessed using the GMDS scale, were similar between infants who received Intralipid® and those who received SMOFLipid®, both in total score and across subscales. The only exception was Scale D (eye and hand coordination), where infants in the SMOFLipid® group achieved significantly higher scores. After applying correction for multiple testing, the statistically significant difference observed in Scale D was confirmed. This domain encompasses fine motor skills, manual dexterity, and visuoperceptual abilities, where a slight but statistically significant difference was noted.

The GMDS scores assessed at 2 years of corrected age were lower than those reported in some comparable cohorts of preterm infants (17, 23). However, in other cohorts (45, 46), GMDS scores may be broadly similar to ours, suggesting variability across studies. These differences could be influenced by unmeasured confounding factors such as parental educational level or other sociodemographic variables that were not considered. Nevertheless, the two study groups in our cohort were comparable to each other, and the similarity of their GMDS outcomes reinforces the conclusion that the type of lipid emulsion used for parenteral nutrition did not significantly influence neurodevelopmental outcomes. Compositional differences between SMOFlipid® and Intralipid® may influence brain development and clinical outcomes. The finding of a better performance in Scale D (eye and hand coordination) may be partially explained by the effects of fish oil-based emulsions on retinal maturation. Yalagala (47) observed improved retinal development in infants of mothers with DHA-rich diets, and Turner et al. (48) demonstrated enhanced retinal function in piglets fed SMOFLipid®. Although Binder et al. (25) did not find significant differences in visual evoked potentials, a trend toward faster neural conduction in the SMOFLipid® group was noted. The absence of similar findings in other studies, such as that by Gallini et al., could reflect the timing of these effects—potentially manifesting later in development—or simply chance, given the small sample sizes and the complexity of neurodevelopmental trajectories in preterm infants.

Given the known influence of omega-3 long-chain polyunsaturated fatty acids on fetal brain development, it is relevant to consider maternal dietary intake as a parallel factor. Maternal dietary supplementation with omega-3 polyunsaturated fatty acids has been shown to modulate fetal oxidative stress and inflammatory status (8), as well as brain development and maturation (44, 45, 47). Bruschi et al. (8) demonstrated that such supplementation in women at risk of preterm delivery was associated with improved redox balance and proteomic signatures associated with neurodevelopmental protection in preterm neonates. Yalagala (47) showed that maternal dietary enrichment with DHA could be a promising strategy to improve brain and retinal health in infants. Similarly, De Bernardo (46) demonstrated that the DHA content of human milk from mothers who supplement DHA during pregnancy remains consistent regardless of gestational age at delivery. However, the impact of different lipid formulations in parenteral nutrition on the neurodevelopment of preterm infants remains controversial (24, 34, 49, 50).

In a recent meta-analysis (48), SMOFlipid® offers a promising option for preterm infants compared to Intralipid®, particularly for reducing cholestasis and PDA, but no differences were found for the prevention of several other neonatal outcomes, including growth, mortality, ROP, and BPD, confirming the findings of a recent Cochrane review (18).

Several studies have reported that the inclusion of omega-3 s may have beneficial effects on neurodevelopment in preterm infants (26, 27). A study by Ottolini et al. (19) reported that very preterm infants supported with SMOFLipid® demonstrated improved regional brain growth and evidence of enhanced white matter microstructural organization and neurobehavioral regulation at term-corrected age.

Neurodevelopmental data of our study were consistent with those of Gallini et al. (17), who found no significant differences at 24 months between infants treated with MLE and those treated with SLE. However, their study focused on anthropometric evaluation at 12 and 24 months CA, short-term neurodevelopmental outcomes at 6 and 12 months CA using the Hammersmith infant neurological examination (HINE), and long-term neurodevelopmental outcomes at 24 months CA using GMDS, while our study expands the analysis to include brain maturation at term-equivalent age using the TMS.

The two cohorts showed no statistically significant differences in anthropometric measures, maternal characteristics, or preterm complications. A particular mention should be made regarding BPD. Previous studies have suggested that different lipid formulations may modulate the pulmonary inflammatory response (22).

Regarding BPD, although the incidence appeared higher in the Intralipid® group, this difference did not reach statistical significance after Benjamini–Hochberg correction (adjusted *p* = 0.26) and is therefore unlikely to have affected neurodevelopmental results. The numerically greater percentage of BPD in the Intralipid® group can be explained by a gradual change in clinical practice during the study period, with reduced use of invasive mechanical ventilation in favor of non-invasive respiratory support—a strategy well documented to lower BPD risk. However, emerging evidence suggests that newer-generation lipid emulsions enriched with omega-3 long-chain polyunsaturated fatty acids, such as SMOFLipid®, may exert anti-inflammatory effects and reduce oxidative stress—both of which are implicated in the pathogenesis of BPD. Several studies have reported a trend toward lower BPD incidence in infants receiving fish oil-containing emulsions, potentially due to their favorable fatty acid profile and antioxidant content (21, 22).

Finally, patients with cerebellar lesions and white matter injuries of any grade were excluded, given their well-established association with adverse neurodevelopmental outcomes in preterm infants (3, 6, 7). Severe hemorrhagic lesions were also excluded, while GMH were retained. Although our recent study have suggested a potential role of low-grade GMH in influencing neurodevelopment (51), there are contrasting data on this subject (52). Moreover, in our cohort, the prevalence of GMH was similarly distributed across groups, making it an unlikely confounding factor in our analysis.

This study has several significant limitations that should be considered. First, its retrospective design inherently limits the ability to establish causal relationships, as data were collected from past records rather than through prospective observation. Additionally, being conducted at a single center may reduce the generalizability of the results to broader populations, as institutional practices, patient demographics, and regional factors may differ significantly from those in other settings. Moreover, the treatment groups were enrolled at different time periods, which could introduce bias due to changes in NICU protocols. However, during this timeframe, the only relevant change was the pharmacological treatment for patent ductus arteriosus (PDA), which shifted from ibuprofen to paracetamol (53)—a modification not expected to influence neurodevelopment at 2 years of corrected age.

Several potential confounders could have influenced brain maturation during the 2-year follow-up, despite our efforts to minimize bias by excluding infants with major brain lesions and severe complications. Although the two groups were comparable in terms of common complications of prematurity, we lacked complete data on important sociodemographic variables such as maternal age at birth, parental educational level, and ethnic background. These factors are well recognized as potential determinants of neurodevelopment in preterm infants after discharge. Since this information was unavailable for all participants, it could not be included in our analysis and may have introduced residual confounding. Furthermore, the sample size was too small to detect clinically relevant differences of 5 points in the GDQ and was also insufficient to identify differences in TMS scores. Based on our calculations, detecting a 5-point difference in GDQ would require approximately 253 infants per group (total ≈ 506), and detecting differences in TMS would require even larger samples. These limitations could result in underestimating potential associations or failing to identify clinically relevant trends. Additionally, maternal dietary data and detailed nutritional intake were not available, which could influence outcomes. These calculations suggest that future prospective trials should either focus on larger clinically meaningful differences or adopt a multicenter design to achieve adequate power.

## 5 Conclusion

Despite integrating brain imaging and clinical follow-up data, the retrospective nature of the study and the change in lipid emulsion use over time indicate that our findings do not allow us to determine the optimal lipid emulsion for preterm infants. This question would require prospective randomized controlled trials to be adequately addressed.

The widespread adoption of MLE by clinicians, despite limited supporting evidence, may reflect the perceived health benefits of fish and olive oil. From a nutritional perspective, soy-based lipids—central to Intralipid®—are not typically prominent in maternal diets, raising questions about their suitability for fetal brain development. Conversely, emerging evidence recommends that SMOFLipid®, enriched with omega-3 long-chain polyunsaturated fatty acids, may reduce inflammation and oxidative stress, potentially lowering brain oxidative stress and BPD risk. Both groups had a median gestational age of 29 weeks—a critical period for myelination (54)—highlighting the importance of optimizing parenteral lipid composition during this vulnerable window, when infants rely primarily on intravenous nutrition as a substitute for placental support. Further research is essential to clarify these associations.

## Data Availability

The raw data supporting the conclusions of this article will be made available by the authors, without undue reservation.
